# A Fibre Bragg Grating Sensor as a Receiver for Acoustic Communications Signals

**DOI:** 10.3390/s110100455

**Published:** 2011-01-04

**Authors:** Graham Wild, Steven Hinckley

**Affiliations:** Optics Research Laboratory, Centre for Communications Engineering Research, School of Engineering, Edith Cowan University, 270 Joondalup Drive, Joondalup 6027 WA, Australia; E-Mail: S.Hinckley@ecu.edu.au

**Keywords:** acoustic sensing, fibre Bragg grating, optical fibre sensing, wireless communications

## Abstract

A Fibre Bragg Grating (FBG) acoustic sensor is used as a receiver for acoustic communications signals. Acoustic transmissions were generated in aluminium and Carbon Fibre Composite (CFC) panels. The FBG receiver was coupled to the bottom surface opposite a piezoelectric transmitter. For the CFC, a second FBG was embedded within the layup for comparison. We show the transfer function, frequency response, and transient response of the acoustic communications channels. In addition, the FBG receiver was used to detect Phase Shift Keying (PSK) communications signals, which was shown to be the most robust method in a highly resonant communications channel.

## 1. Introduction

In this paper, we demonstrate the use of a Fibre Bragg Grating (FBG) acoustic sensor as a receiver for acoustic communications signals through elastic solids. The effectiveness of the FBG receiver is shown side by side a piezoelectric receiver for direct comparison. Two different materials were used as the communications media: aluminium (Al) and Carbon Fibre Composite (CFC) panels. These materials were chosen simply due to their prevalence in the aerospace industry. The communications channels were characterized in terms of their transfer function, frequency response, and transient response (at resonance). In addition, sample communications signals were also tested for each of the channels. We show results of the three basic digital modulation methods: Amplitude Shift Keying (ASK), specifically, On Off Keying (OOK), Frequency Shift Keying (FSK), and Phase Shift Keying (PSK).

Acoustic communications is a wireless communications method commonly used in underwater applications [1]. Acoustic communications has also been proposed to communicate with *in vivo* biomedical devices [2]. Acoustic communications also has applications for data transmissions through elastic solids; in particular EMI hardened structures [3] and pressurized structures [4], where wireless RF or wired communications are not possible. In Structural Health Monitoring (SHM) systems that utilize acoustic based Non-Destructive Evaluation (NDE), acoustic actuators and receivers are readily available, hence, acoustic communications can be utilized.

The detection of Acoustic Emissions (AEs) is a current area of research for Aerospace Vehicle SHM, with applications to the detection and monitoring of micrometeorite and space debris impacts [5]. Similarly, this type of SHM system also has applications in aircraft, where bird strikes and tool drops are a concern, and where the delamination of composites is a problem [6]. Typically, a SHM system to detect acoustic emissions and/or strain is intended to do so in real time. However, secondary damage may result from the initial impact or strain. This damage may include surface pitting, erosion, or cracking. These types of secondary damage may not be detectable, and hence may not be able to be monitored by the embedded SHM system, even with the inclusion of more advance techniques, such as Acousto-Ultrasonics (AU). To overcome this, the use of robotic agents to perform further NDE has been suggested [7]. The integration of robotic NDE into SHM would enable the detection and monitoring of a wider variety of damage. Information then collected by the robotic agent needs to be communicated to the SHM system, so it can used utilized. Acoustic communication is then an ideal method to communicate information from an autonomous agent to a SHM system.

As Optical Fibre Sensors (OFSs), FBGs offer several advantages that make them of interest to sensing areas, especially SHM [8]. The most significant of these advantages include reduced size and weight, immunity to electromagnetic interference, and most significantly, the versatility of FBGs to detect different measurands. For SHM, a FBG system can be used to detect Acoustic Emissions (AEs), actively generated AU signals, dynamic strain (e.g., vibration), static strain (e.g., load monitoring), and corrosion, as well as a variety of other measurands. Optical fibre sensors, specifically FBGs, have been utilized extensively for the detection of acoustic signals [9]. FBGs have been investigated for use in SHM of both composite structures [10] and aluminium structures [11].

## 2. Theory

### 2.1. Sensor

FBGs are well known passive devices utilized extensively in optical fibre communications and sensing [12]. The periodic grating acts as a filter, reflecting a narrow wavelength range, centered about a peak wavelength. This wavelength, known as the Bragg wavelength (*λ_B_*), is given by [12]:

(1) $$\lambda_{B}=2n\Lambda$$

where *n* is the average refractive index of the grating and Λ is the grating period.

Any measurand that has the ability to affect either the refractive index or the grating period can be measured using an FBG as a sensor. Specifically, an FBG is sensitive to strain and temperature. The relative change in the Bragg wavelength (Δ*λ_B_*) as a function of the applied strain (*ε*), can then be expressed as [12]:

(2) $${\Delta \lambda }_{B}=\lambda_{B}\left[\varepsilon \left(1-\frac{n^{2}}{2}\left[p_{12}-\nu \left(p_{12}+p_{11}\right)\right]\right)+\Delta T\left(\alpha +\frac{1}{n}\frac{\mathrm{dn}}{\mathrm{dT}}\right)\right]$$

where *v* is Poisons’ ratio, *p*_12_ and *p*_11_ are the strain optic coefficients.

**Figure 1.**
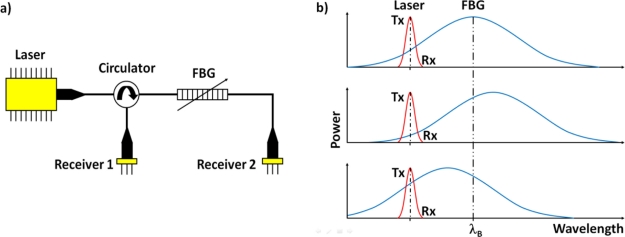
**(a)** The Optical circuit of the TRDS. **(b)** The operation of the TRDS, showing the spectrum of the FBG and the laser, with no applied strain (top), positive strain (middle), and negative strain (bottom), indicating the increase and decrease in the Tx and Rx signals.

Typically, power detection is used to convert the shift in the Bragg wavelength to an intensity change [13]. Here, one side of the FBGs spectral reflectivity curve is used as a linear filter. A narrow band laser source, centered about the 3 dB point, is then intensity modulated as the applied signal shifts the filter response. The modulation that occurs to the reflected signal also occurs to the transmitted signal [14]. When the change in the reflected optical power is positive, the change in transmitted optical power is negative, and vice versa. Since one signal is positive, and the other is negative, the transmitted (Tx) and reflected (Rx) signals can be differentially amplified, giving increased signal strength. The configuration of the optical circuit for this Transmit Reflect Detection System (TRDS) is shown in Figure 1(a), with Figure 1(b) illustrating the principle of operation. The TRDS has previously been used for the detection of AU signals [15].

### 2.2. Communications

Due to the properties of the communications channel, only digital encoding methods have been investigated. The primary benefit of which is increased signal fidelity. The three basic digital encoding methods used include Amplitude Shift Keying (ASK), Frequency Shift Keying (FSK) and Phase Shift Keying (PSK). For the purpose of concept demonstration, only binary keying methods were utilized.

#### Amplitude shift keying (ASK):

In ASK, the digital information is encoded onto the analogue carrier as a time varying amplitude signal. The simplest form of ASK is OOK, where a ‘1’ is represented by the amplitude function being maximum (on), and a ‘0’ is represented by the amplitude function being zero (off). The OOK signal will have the form:

(3) $$f\left(t\right)=A(t)\;\text{cos}\left(2\pi f_{c}t\right)$$

where *f*_c_ is the carrier frequency, and:

(4) $$A(t)=\{\begin{array}{ll} 0 & \mathrm{for}\;\;\mathrm{data}=0\\ A & \mathrm{for}\;\;\mathrm{data}=1 \end{array}$$

**Figure 2.**
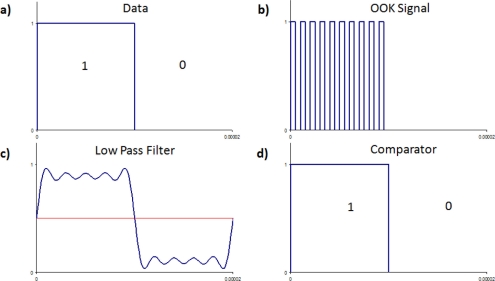
Decoding an amplitude shift keying signal, **(a)** the digital data to be transmitted, **(b)** the on-off keying signal, **(c)** the rectified and low pass filtered signal, **(d)** digital information recovered after a comparator.

Figure 2(a) shows the data to be transmitted defined by Equation (4), and Figure 2(b) shows the on-off keying signal as defined by Equation (3). OOK is decoded by rectifying the received signal and then using a low pass filter that has a cutoff frequency above the data rate, but below the carrier frequency, Figure 2(c). This removes the carrier wave component (cos(2*πf_c_t*)) and recovers the envelope. The signal is finally passed through a comparator to recover the digital information signal, A(t), as shown in Figure 2(d).

#### Frequency shift keying (FSK):

In FSK, the digital information is encoded onto the analogue carrier as a time varying signal of the frequency. In binary FSK, two frequencies are used; one frequency represents a digital ‘1’ and the second represents a digital ‘0’. FSK can be thought of as two interweaved OOK signals with different carrier frequencies. This means that a similar non-coherent decoding method can be used to recover the digital information. However, the advantage FSK has over ASK is lost in this way. To maintain the independence of the signal from amplitude variations, a coherent detection method is used with continuous-phase FSK. Here, the received signal is split into two separate, but identical signals; each of the form:

(5) $$f\left(t\right)=A_{0}\;\text{cos}\left(2\pi f_{c}\left(t\right)t\right)$$

where:

(6) $$f_{c}(t)=\{\begin{array}{ll} f_{1} & \mathrm{for}\;\;\mathrm{data}=0\\ f_{2} & \mathrm{for}\;\;\mathrm{data}=1 \end{array}$$

**Figure 3.**
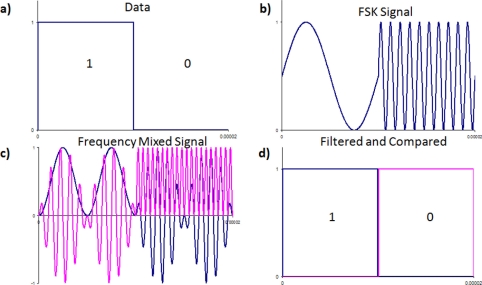
Decoding a frequency shift keying signal, **(a)** the digital data to be transmitted, **(b)** the frequency shift keying signal, **(c)** the frequency mixed signal, **(d)** digital information recovered after filtering and comparing.

The data transmitted (Figure 3(a)) is encoded as two separate frequencies in the signal as defined by Equation (4). The resultant FSK signal is shown in Figure 3(b). The phase of the signal is maintained at the bit change; hence, the modulation is referred to as continuous-phase FSK.

The two signals are each multiplying with a synchronous cosine, one with frequency *f*_1_, the other with frequency *f*_2_, shown in Figure 3(c). This shifts the signal to zero and 2*f*_n_, that is, one of the data bits has no DC offset, while the second bit has a DC offset. A low pass filter is used to remove the 2*f*_n_ component from each signal. When filtered, the lack of an offset will result in a zero, while an offset will give a one. The two filtered signals are then compared to each other to recover the digital information. The recovered signal after the filter and comparator is shown in Figure 3(d).

#### Phase shift keying (PSK):

In PSK, the digital information is encoded onto the analogue carrier as a time varying signal of the phase. The PSK signal is defined as:

(7) $$f\left(t\right)=A_{0}\;\text{cos}\left(2\pi f_{c}t+\phi \left(t\right)\right)$$

where:

(8) $$\phi (t)=\{\begin{array}{ll} -90 & \mathrm{for}\;\;\mathrm{data}=0\\ 90 & \mathrm{for}\;\;\mathrm{data}=1 \end{array}$$

**Figure 4.**
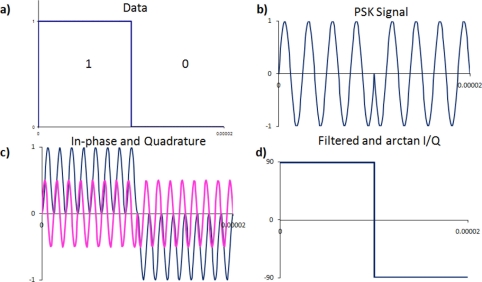
Decoding a phase shift keying signal, **(a)** the digital data to be transmitted, **(b)** the phase shift keying signal, **(c)** the in-phase and quadrature components, **(d)** digital information recovered after filtering and comparing.

The digital information from Equation (8) is shown in Figure 4(a), and the PSK signal with the 180 degree phase shift, is shown in Figure 4(b).

Decoding PSK uses some simple mathematics to retrieve the phase information The PSK signal, Equation (7), is multiplied by a synchronous sine and cosine, giving:

(9) $$\begin{array}{cc} h(t) & =A_{0}\;\text{cos}\left(2\pi f_{c}t+\phi (t)\right)\times \text{sin}\left(2\pi f_{c}t\right)\\ & =\frac{A_{0}}{2}\left[\text{sin}\left(\left(4\pi f_{c}t+\phi (t)\right)\right)+\text{sin}\left(\phi (t)\right)\right] \end{array}$$

and:

(10) $$\begin{array}{cc} g(t) & =A_{0}\;\text{cos}\left(2\pi f_{c}t+\phi (t)\right)\times \text{cos}\left(2\pi f_{c}t\right)\\ & =\frac{A_{0}}{2}\left[\text{cos}\left(\phi \right)+\text{cos}\left(\left(4\pi f_{c}t+\phi (t)\right)\right)\right] \end{array}$$

These two components are called the in-phase (I) and quadrature (Q) components. Both I and Q contain high and low frequency components, where the low frequency component is the sine or cosine of the time dependent phase. The components, I and Q, are shown in Figure 4(c). When filtered, the mixed signals show that the in-phase component has a positive value for the first bit, then a negative value for the second bit, while the quadrature component has a value of zero. Using a low pass filter the high frequency components are removed, leaving only the phase component:

(11) $$\begin{array}{l} h^{\prime }(t)=\frac{A_{0}}{2}\text{sin}\left(\phi (t)\right)\\ g^{\prime }(t)=\frac{A_{0}}{2}\;\text{cos}\left(\phi (t)\right) \end{array}$$

Then by taking the arctangent of I on Q, the time dependent phase information is recovered:

(12) $$\begin{array}{ll} y(t) & =\text{arctan}\left(\frac{h^{\prime }(t)}{g^{\prime }(t)}\right)\\ & =\text{arctan}\left(\frac{\text{sin}(\phi (t))}{\text{cos}(\phi (t))}\right)\\ & =\text{arctan}\left(\text{tan}(\phi (t))\right)\\ & =\phi (t) \end{array}$$

The recovered phase information is shown in Figure 4(d). The filter used was a raised cosine filter [16].

## 3. Methodology

### 3.1. Experimental Setup

**Figure 5.**
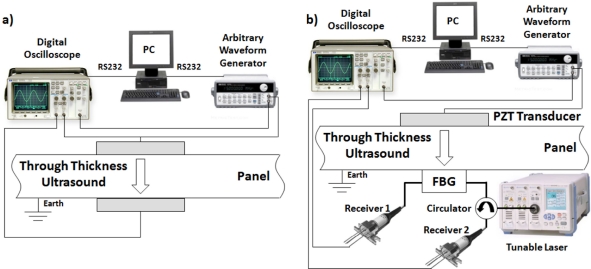
The experimental setup using, **(a)** the PZT, and **(b)** the FBG receiver.

For the purpose of establishing a base line, the first system tested used both a piezoelectric transmitter and a piezoelectric receiver. The PZT transducers used were unbacked, and coupled to opposite facets of the panels using an acoustic coupling gel. They had a thickness of 2.1 millimeters (giving a resonant frequency of approximately 1 MHz), and a diameter of 20 millimeters (giving a resonant frequency of approximately 100 kHz). The configuration of the PZT to PZT acoustic communications channel is shown in Figure 5(a).

Next, the FBG receiver was used in place of the PZT receiver. The FBG receiver included a tunable laser, circulator, and two photoreceivers. The first FBG configuration, shown in Figure 5(b), had the FBG coupled opposite the PZT transmitter. Again acoustic coupling gel was used to couple the FBG to the panels. The second FBG configuration, not illustrated, had the FBG located in-between the second and third layers of a four layer CFC stack.

In total, five configurations were tested: PZT transmitter, Al medium, PZT receiver (Al-PZT); PZT transmitter, CFC medium, PZT receiver (CFC-PZT); PZT transmitter, Al medium, FBG receiver (Al-FBG); PZT transmitter, CFC medium, coupled FBG receiver (CFC-cFBG); and, PZT transmitter, CFC medium, embedded FBG receiver (CFC-eFBG).

The FBG with Al-FBG configuration has previously been used for the detection of continuous wave acoustic signals [15]. The configuration was sensitive enough to detect the acoustic emission from a lead pencil break test [17].

### 3.2. Acoustic Transmissions

All five configurations were used to test a number of different quantities with regards to the ability to receive actively generated acoustic signals. These included transfer function to give the sensitivity (output voltage as a function of PZT driving voltage), frequency response, and transient response at the resonant frequency. Also, a sample communications signal was generated using PSK.

First, the frequency responses of the communications channels were measured to determine the resonate frequencies. The function generator was set to give a continuous sine wave at maximum voltage, 10 V peak. The frequency was then varied from 10 kHz to 1.5 MHz. Values were recorded every 10 kHz, except at significant peaks, where values were recorded at every kilohertz.

Second, the transfer function (also referred to as the transfer characteristic) of the systems was measured. The function generator driving the PZT transmitter was set to give a continuous sine wave at the resonant frequency of each system. The amplitude was then varied from 1 V to 10 V. Output voltage values were recorded at 1 V increments.

Finally, the transient response of the systems was investigated. A low rate sine wave burst at resonance was used, with 100 cycles. The trailing signal was also examined to determine if it would have any adverse effects on the performance of the communications channel.

### 3.3. Acoustic Communications

After the fundamental properties of each communications channel was investigated, acoustic communications signals were tested. The ASK and FSK communications signals were generated directly with the waveform generator, using the burst functionality to simulate an ASK signal, and the dedicated FSK functionality. The PSK signal was generated in the Waveform Editor software for the waveform generator. The signal was then flashed to the device via the computer interface. The waveform generated consisted of a sine wave carrier, with data rates of 1/100 the carrier frequency. All of the signals were recorded on a digital oscilloscope, and downloaded to a PC, where the decoding algorithms were implemented in Matlab (The Mathwork Inc). For comparison, the basic ASK, FSK, and PSK signals were compared using both the Al-PZT channel and the Al-FBG channel to show the difference using the FBG as the receiver. Next, the Al-FBG channel was compared with the CFC-eFBG channel, to show the effect of changing the communications medium on the received communications signal. The CFC-cFBG panel was not used due to the performance of the eFBG.

## 4. Results and Discussion

**Figure 6.**
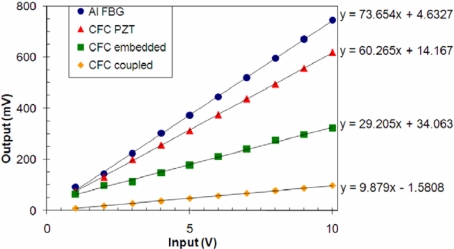
Transfer functions of the acoustic communications channels.

The transfer functions for four of the five configurations tested are shown in Figure 6. The Al-PZT configuration is not shown due to the significantly larger efficiency at the through thickness resonance, with a sensitivity of 882.8 mV/V. All transfer functions are linear, and show that significant signal strengths are achievable in all configurations, even with no amplification.

Clearly the Al channel shows greater signal strengths, even when using the FBG as the receiver. The reduction in signal strength when using the CFC is most likely due to the anisotropic nature of the material. The interesting result comes from comparing the difference in gain between the Al configurations with the difference between the CFC configurations. There is a reduction in sensitivity by a factor of 5.5 from the Al-PZT to the Al-FBG, at 100 kHz. There is only a reduction by a factor of 2 when using the eFBG compared to the PZT. The reduction in signal strength from the PZT to cFBG is a factor of 6, which is very similar to the Al value. This significant improvement in performance of the FBG receiver when embedded in the CFC is due to the fact that the acoustic coupling gel, although viscous, does not transmit the strain effectively. If the PZT receivers were bonded to the panels, we would expect to see a similar improvement in their signal strength.

**Figure 7.**
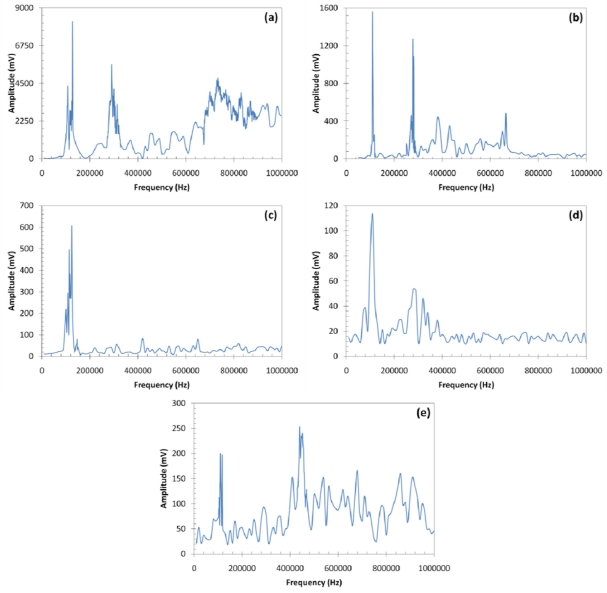
Frequency response of the acoustic communications channels, **(a)** Al-PZT, **(b)** Al-FBG, **(c)** CFC-PZT, **(d)** CFC-cFBG, and **(e)** CFC-eFBG.

The frequency responses of all five configurations are shown in Figure 7. All curves show a low frequency resonance, about 100 kHz, which corresponds to the first radial mode of the PZT transducer, with a diameter of 20 mm. The most interesting feature of the frequency response curves is the primary peak at 440 kHz for the CFC-eFBG channel, which is not a significant feature in any other curve. This peak is broad which is good for communications purposes, since data rate is related to bandwidth.

**Figure 8.**
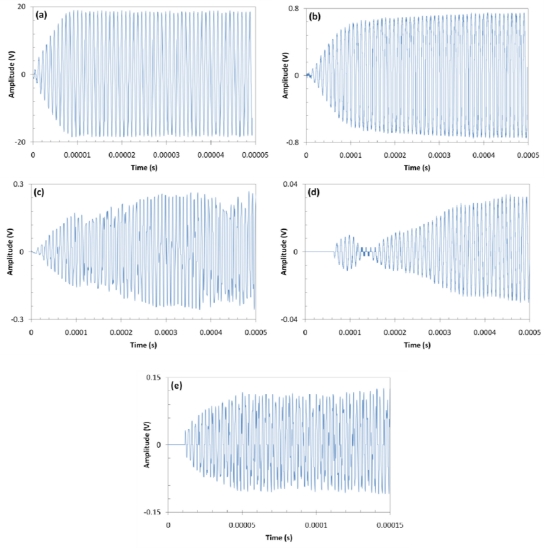
Transient response of the acoustic communications channels, **(a)** Al-PZT, **(b)** Al-FBG, **(c)** CFC-PZT, **(d)** CFC-cFBG, and **(e)** CFC-eFBG.

The transient response of the five communications channels are shown in Figure 8. The three CFC transient responses are not as smooth as the Al channel. The interesting feature is that the number of cycles required to reach steady state for the CFC-eFBG is less than for the CFC-PZT. This can be explained by the broadness of the 440 kHz peak in the eFBG spectrum compared to the narrower 100 kHz peak for the PZT. The improved frequency response of the eFBG receivers is likely due to the greater sensitivity to shear strain with the FBG in the epoxy resin layer of the CFC layup.

**Figure 9.**
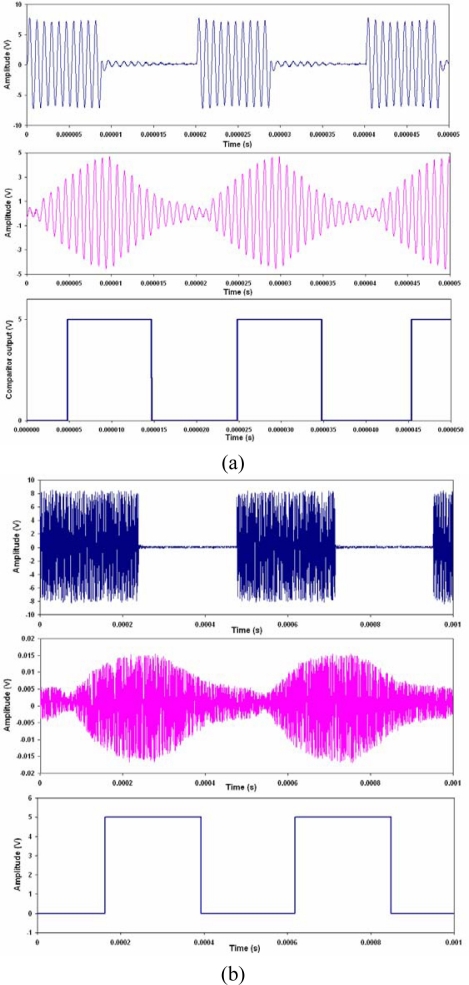
ASK OOK communications signals for **(a)** Al-PZT, **(b)** Al-FBG; showing transmitted **(top)**, received **(middle)** and decoded **(bottom)**.

**Figure 10.**
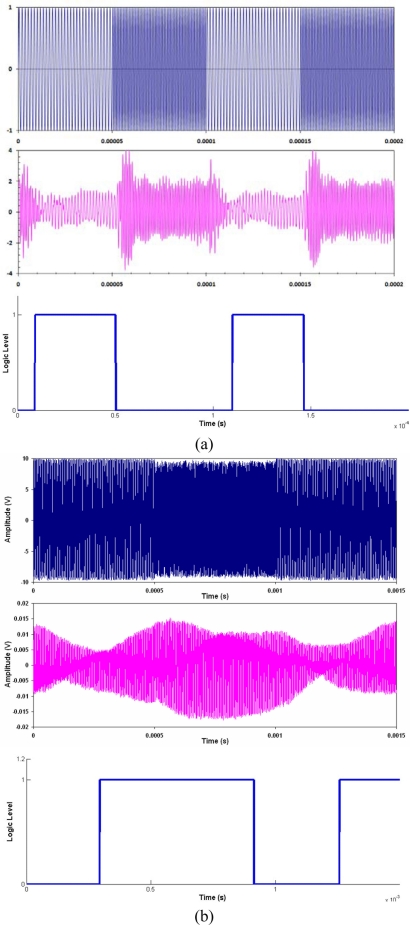
FSK communications signals for **(a)** Al-PZT, **(b)** Al-FBG, showing transmitted **(top)**, received **(middle)** and decoded **(bottom)**.

**Figure 11.**
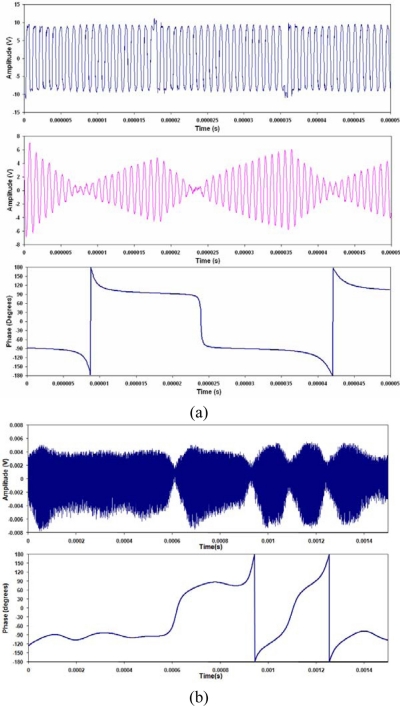
PSK communications signals for **(a)** Al-PZT, **(b)** Al-FBG; showing Transmitted **(top)**, received **(middle)** and decoded **(bottom)**. Note: transmitted signal for FBG PSK signal shows no discernable information so this has been omitted.

**Figure 12.**
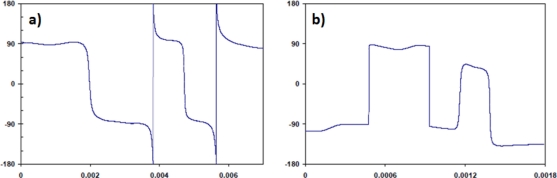
PSK communications signals for **(a)** Al-FBG and **(b)** CFC-eFBG.

Figures 9 to 12 show the communications signals. All the graphs for the same channel configuration are shown on the same time scale, and aligned to show any effects of the channel. In particular, any time delays between switching can clearly be seen. Figure 9 shows the ASK signals, Figure 10 shows the FSK signals, and Figure 11 shows the PSK signals, comparing the PZT receiver to the FBG receiver. Figure 12 shows the comparison between the aluminium and carbon fibre composite channels using the FBG receiver.

The resonant nature of the communications channels means that the symmetry of the ASK signals depended on the value chosen for the comparator. Since the data rate achievable will typically be limited to kilobits, oversampling would be possible in any practical implementation. This would enable a more advanced decision making method to be used, as well as tracking any variation in the amplitude over time. The implementation of ASK, in particular OOK is feasible. When utilizing Lamb waves, OOK may in fact prove more effective than PSK, depending on the geometry of the communications medium, *i.e.*, the structure.

In general, all of the communications signal changes, *i.e.*, switching the amplitude, frequency, or phase, have the same effect relative to the PZT transmitter. All three methods appear as one signal being switched off, and then a second signal being switched on, due to the resonant nature of the channel. The transient response is therefore the limiting factor for the data rate achievable.

When using ASK, if the number of cycles used in a single bit is much less than the number required to reach steady state, errors may be generated after a long sequence of 1’s, when the steady state has been reached. This is due to the fact that the system needs to ‘ring down’ from a larger amplitude. A solution to this problem may be to use coding methods other than Non-Return to Zero (NRZ). Manchester coding for example would ensure that maximum amplitude is reached in a set period, as each single bit is replaced by either a ‘01’ or a ‘10’. This could also be achieved with other mBnB coding, such as 2B3B. The only condition is that no combination of input bits can be replaced by a sequence of 1’s, hence, preventing excessive ‘ringing’ of the system.

The same conclusion for ASK applies to the non-coherent and coherent detection of FSK. In addition, the variation of the transient response with frequency represents a limiting factor for coherent detection. The transient response for both frequencies used in the FSK signals need to be similar. This will ensure that as the signal switches, the bit with one frequency is decaying, and the bit with the second frequency is growing. Ideally, the two should cross at the transition point of the data stream, with equal amplitude. This would require a scaling factor between the two signals. The result of not using a scaling factor is shown in Figure 10, where the data bits are asymmetric.

The random fluctuations mentioned above may have significant problems for FSK signals, specifically if the fluctuations affect different frequencies in different ways. That is, *f*_1_ may decrease in overall amplitude and *f*_2_ may increase in overall amplitude. This could result in the generation of errors. Also, continuous-phase FSK, and more specifically Minimal Shift Keying, MSK (where *f*_2_ = 2*f*_1_), was implemented for the FSK signals. However, the use of MSK was limited given the asymmetric frequency response, and was used for the PZT-Al-PZT channel, while only continuous-phase FSK was used for the PZT-Al-FBG channel. The resonant nature of the communications channel, with different transient response times for different frequencies could affect the implementation of the successful coherent demodulation of the FSK signals. In general, the ability to practically implement FSK is limited.

PSK represents the most effective and reliable method of transmitting data through any of the communications channels. Any variations that result in changes to the amplitude of the signal will in essence have little effect on the phase of the signal. PSK also represents the most likely method to be improved beyond the limit of the transient response. Higher data rates could be achieved if the direction of the phase changes could be chosen. That is, instead of the phase changing in a random direction, from −90° to 90° via 0° or 180° (as seen in the phase diagrams of Figure 11), the phase was forced to switch from −90° to 90° via 0° all the time (as seen in Figure 12(b)), the levels, −90° or 90° would no longer be needed. Smaller values could be used, e.g., −45° or 45°, which could effectively double the data rate with the same channel characteristics.

## 5. Conclusions

In conclusion, we have successfully demonstrated the use of a FBG as a receiver for wireless acoustic communications through elastic solids. The elastic solids used included both aluminium and carbon fibre composite panels. The FBG receiver was compared with a piezoelectric receiver. The performance of the different communications channels were measured, in terms of frequency response, transfer function, and transient response. Simple phase shift keying digital communications signals were also successfully decoded for each of the communications channels.

Generally the use of the PZT receiver appears to have greater signal strength in all cases. However, this is due to the choice of circuit components used in the TRDS. That is, the maximum signal out is limited to 1 V for the FBG receiver, while the maximum signal for the PZT receiver is 10 V. Taking in this consideration, the FBG receiver only gives a slightly smaller sensitivity when using the Al. For the CFC, the FBG actually outperforms the PZT; in particular, the frequency response of the FBG is much broader. Clearly, the FBG acoustic sensor can be successfully used as a receiver for acoustic communications signals, in addition to being able to detect other mechanical waves. When embedded within a CFC, the FBG sensor offers greater sensitivity and frequency response, and in general, the FBG offers the versatility to detect other quantities, specifically, static strain.

## References

[1] Chitre M., Shahabodeen S., Stojanovic M. (2008). Underwater Acoustic Communications and Networking: Recent Advances and Future Challenges. Mar. Technol. Soc. J.

[2] Kawanabe H., Katane T., Saotome H., Saito O., Kobayashi K. (2001). Power and Information Transmission to Implanted Medical Device Using Ultrasonic. Jpn. J. Appl. Phys.

[3] Wild G. (2005). Design and Evaluation of an Electro-Acoustic Communications Channel for Use by Autonomous Agents in the Structural Health Monitoring of Ageless Aerospace Vehicles.

[4] Murphy T.L. (2005). Ultrasonic Digital Communications System for a Steel Wall Multipath Channel: Method and Results.

[5] Price D.C., Scott D.A., Edwards G.C., Batten A., Farmer A.J., Hedley M., Johnson M.E., Lewis C.J., Poulton C.T., Prokopenko M., Valencia P., Wang P., Chang F.K. (2003). An Integrated Health Monitoring System for an Ageless Aerospace Vehicle. Structural Health Monitoring 2003: From Diagnostics & Prognostics to Structural Health Management.

[6] Staszewski W., Boller C., Tomlison G (2004). Health Monitoring of Aerospace Structures: Smart Sensor Technologies and Signal Processing.

[7] Bahr B. Automated Inspection for Aging Aircraft.

[8] Measures R.M. (2001). Structural Monitoring with Fibre Optic Technology.

[9] Wild G., Hinckley S. (2008). Acousto-Ultrasonic Optical Fibre Sensors: Overview and State-of-the-Art. IEEE Sens. J.

[10] Takeda N., Okabe Y., Kuwahara J., Kojima S., Ogisu T. (2005). Development of Smart Composite Structures with Small-Diameter Fibre Bragg Grating Sensors for Damage Detection: Quantitative Evaluation of Delamination Length in CFRP Laminates Using Lamb Wave Sensing. Composites Sci. Technol.

[11] Betz D.C., Thursby G., Culshaw B., Staszewski W.J. (2007). Structural Damage Location with Fibre Bragg Grating Rosettes and Lamb Waves. Struct. Health Monit.

[12] Andreas O., Kalli K (1999). Fibre Bragg Grating: Fundamentals and Applications in Telecommunications and Sensing.

[13] Webb D.J., Surowiec J., Sweeney M., Jackson D.A., Gavrilov L.R., Hand J.W., Zhang L., Bennion I. Miniature Fibre Optic Ultrasonic Probe.

[14] Takahashi N., Hirose A., Takahashi S. (1997). Underwater Acoustic Sensor with Fibre Bragg Grating. Opt. Rev.

[15] Wild G., Hinckley S. (2010). Spatial Performance of Acousto-Ultrasonic Fibre Bragg Grating Sensor. IEEE Sens. J.

[16] Proakis J., Salehi M (1994). Communication Systems Engineering.

[17] Wild G., Hinckley S., Mukhopadhyay S.C., Gupta G.S. (2008). A Transmit Reflect Detection System for Fibre Bragg Grating Acoustic Emission and Transmission Sensors. Lecture Notes in Electrical Engineering—Smart Sensors and Sensing Technology.

